# One-year clinical and radiographic evaluation of young permanent molars treated with brix 3000 vs. papacárie duo: a randomized controlled clinical trial

**DOI:** 10.1186/s12903-025-06715-7

**Published:** 2025-09-23

**Authors:** Passant H. Hassanein, Abdel Wahab M. Samaha, Azza S. Zakaria, Dalia M. Talaat

**Affiliations:** 1 Assistant Lecturer at Pediatric Dentistry and Dental Public Health Department, Faculty of Dentistry, Alexandria University, Alexandria, Egypt; 2 Professor of Pediatric Dentistry and Dental Public Health Department, Faculty of Dentistry, Alexandria University, Alexandria, Egypt; 3 Professor of Microbiology and Immunology Department, Faculty of Pharmacy, Alexandria University, Alexandria, Egypt

**Keywords:** Papain, Dental atraumatic restorative treatment, Minimally invasive, Children, Antimicrobial efficacy, Oral health

## Abstract

**Background:**

Managing deep carious lesions in immature permanent molars presents a clinical challenge. Minimally invasive caries removal helps preserve apexogenesis by minimizing tissue loss. This study evaluated the effectiveness of two chemo-mechanical caries removal (CMCR) agents compared to hand excavation alone in young permanent molars.

**Methods:**

A three-arm randomized clinical trial included 108 children (8–10 years) with carious first permanent molars. Participants were assigned to: Group I (ART with Brix 3000), Group II (ART with Papacárie Duo), or Group III (ART with hand excavation). Dentin samples were collected before and after caries removal to assess *S. mutans* and *Lactobacilli* counts (CFU/ml). Time for caries removal and pain perception were recorded. Clinical and radiographic outcomes were evaluated at 3, 6, and 12 months. OHRQoL was assessed using CPQ8-10 at baseline and during the follow up.

**Results:**

The control group showed significantly shorter caries removal time (5.3 min) than Brix 3000 (11.3 min) and Papacárie Duo (12.1 min) (*P* < 0.01). However, higher pain scores were reported with hand excavation compared to Brix 3000 (*P* = 0.003) and Papacárie Duo (*P* < 0.001). Both within- and between-group comparisons showed significant reductions in *S. mutans* and *Lactobacilli*, with greater bacterial reduction in CMCR groups than ART alone (*P* < 0.001). CMCR groups had significantly higher restoration success rates than the control at 6 and 12 months (*P* = 0.02), and radiographic success was also greater at 12 months (*P* < 0.05). CPQ8-10 scores improved post-treatment across all groups (*P* < 0.001), with a notable difference at 6 months (*P* = 0.008).

**Conclusions:**

Brix 3000 and Papacárie Duo were more effective than hand excavation in reducing cariogenic bacteria, minimizing pain, and enhancing restoration longevity in immature permanent molars. Both CMCR methods supported favorable clinical, radiographic, and quality-of-life outcomes over one year.

**Trial registration:**

This study was registered in ClinicalTrails.gov (NCT05983900) 09/06/2023, https://clinicaltrials.gov/ct2/show/NCT05983900.

**Supplementary Information:**

The online version contains supplementary material available at 10.1186/s12903-025-06715-7.

## Background

Managing deep carious lesions in pediatric patients presents significant challenges, with a critical focus on preserving pulp vitality, especially in asymptomatic permanent teeth. Traditional caries removal techniques often lead to the loss of healthy tooth structure, which can result in pulp exposure and potentially necessitate endodontic treatment [1]. Additionally, apexification procedures may fail to achieve proper root development, increasing the risk of tooth fractures [2]. Over the past few decades, the management of dental caries has undergone a paradigm shift, transitioning from curative interventions to a greater emphasis on preventive strategies. Advances in dental caries research and materials science have led to the adoption of minimally invasive techniques, including selective caries removal by hand instrument, laser, air abrasion, ultrasound, and Chemo-mechanical Caries Removal (CMCR) [3].

COVID-19 pandemic, declared by the World Health Organization in early 2020, has further impacted dental practice. Aerosol-generating procedures, common in traditional dental treatments, pose significant risks for transmitting respiratory infections, including COVID-19, thus endangering dental professionals [4]. As a result, there is increased hesitancy towards using rotary instruments known for aerosol production [5]. In this context, CMCR agents have gained prominence as a safer alternative, as they do not generate aerosols [6]. CMCR agents act selectively removing only infected tissue and eliminates the need for local anesthesia, which is particularly advantageous for patients with dental anxiety or fear [7]. Although local anesthesia is commonly linked with contemporary pain-free dentistry, certain procedures can be effectively conducted without it, which is a significant advantage of the chemo-mechanical approach [8].

CMCR agents are designed to eliminate infected dentin while preserving affected dentin that can be naturally or synthetically remineralized [9]. The challenge in developing these agents lies in ensuring their clinical efficacy and safety, particularly in terms of compatibility with healthy tissue and the pulp [10]. CMCR agents are primarily classified into two types: sodium hypochlorite-based (e.g., Caridex, Carisolv) and papain-based (e.g., Papacárie Duo, Carie-Care, Brix 3000) [6]. The first CMCR agent, GK101, used 5% sodium hypochlorite but was found to be corrosive to healthy tissues [11]. It was subsequently replaced by Caridex, which, while more stable, was criticized for its slow caries dissolution [11]. Carisolv, developed as an alternative, incorporates amino acids and a reduced amount of sodium hypochlorite [11]. Papain, a proteolytic enzyme similar to human pepsin, exhibits bactericidal and bacteriostatic properties [12, 13] Papacárie, introduced in 2003, contains papain at a concentration of 6000 U/mg combined with chloramine as an antimicrobial agent. Brix 3000, released in 2012, represents an advanced formulation with a higher concentration of papain (30,000 U/mg) [14]. Unlike Papacárie, Brix 3000 incorporates toluidine blue for its antimicrobial effect and omits chloramine to improve tissue compatibility and safety [15].

Clinical studies investigating Brix 3000 and Papacárie Duo in young permanent teeth are scarce in the literature [16, 17]. Furthermore, there is a clear gap in comprehensive research regarding the long-term effects of these treatments, particularly in pediatric populations. Clinical follow-up is essential for monitoring treatment outcomes over time, which is crucial for understanding the long-term impact and sustainability of these interventions [18]. Because residual bacteria in deep carious lesions may compromise pulp vitality and lead to further disease progression, assessing the antibacterial effectiveness of these agents is critical especially when aiming to preserve pulp health in young patient [19, 20]. While counts below 10² CFUs/mL for *Streptococcus mutans* (*S. mutans*) and *Lactobacilli* have been deemed acceptable, molecular techniques suggest that traditional culture methods might underestimate bacterial presence [21, 22]. In addition, evaluating oral health-related quality of life (OHRQoL) offers valuable insight into how these treatments affect children’s daily functioning and overall well-being. Therefore, this study aims to address these gaps by assessing the clinical, radiographic, microbiological, and OHRQoL outcomes of Brix 3000 and Papacárie Duo, compared with hand excavation.

The tested null hypothesis stated that there is no significant difference between the CMCR agents and ART using hand excavation without any chemical agents, regarding the time required for caries removal, pain experienced during the procedure, antimicrobial efficacy, clinical and radiographic success during follow-up and their impact on OHRQoL in pediatric patients.

## Methods

### Study design

The present study is a randomized controlled clinical trial with a three-arm parallel design, registered at ClinicalTrials.gov (NCT05983900) on 09/06/2023. It was conducted in Pediatric Dentistry and Dental Public Health Department, Faculty of Dentistry, Alexandria University, Egypt. The study protocol was approved by the Institutional Review Boards (IRB) of Research Ethics Committee, Faculty of Dentistry, Alexandria University, Egypt (IRB NO 00010556-IORG 0008839-12/2022). All procedures were followed in compliance with the Helsinki Declaration and subsequent amendments. The study report follows the protocol defined by the Consolidated Standards of Reporting Trials Statement (CONSORT) checklist [23].

### Sample size estimation

The sample size was estimated assuming a 5% alpha error and 80% study power. The overall success rate after 1 year was 95% for chemo-mechanical caries removal [24], whereas it was 67% for hand excavation [25]. Based on the difference between independent proportions, the sample size was calculated to be 30 patients per group, increasing to 36 patients to make up for lost follow-up cases. Total sample = number per group x number of groups = 36 × 3 = 108 patients.

### Patient recruitment

Two hundred with a behavior rating of 3 or 4 according to Frankl et al. [26] aged 8–10 years were assessed for eligibility in the study. One hundred and eight patients with one deep carious occlusal lesion in the first permanent molar with a score of 5 or 6 according to the International Detection and Assessment System II (ICDAS II), detected by visual-tactile inspection to assess lesion severity [27] were included in the study. This to ensure that CMCR agents could reach the carious dentin without drills or cavity modification. While children who reported spontaneous or elicited pain from caries or showing any signs of pulpal infection, swelling, abscess or exhibited uncooperative behavior (Frankl scores 1 or 2) were excluded from the study. Informed consent from parents and/or legal guardians, as well as the children’s approval to participate in the study, were acquired.

### Randomization technique, grouping, and allocation concealment

108 children were randomly assigned to three groups. Each group consisted of 36 patients

 Test group I: ART with Brix 3000.

 Test group II: ART with Papacárie Duo.

 Control group: ART (hand excavation without the use of any chemical agents).

Participants were assigned at random from a computer-generated list of numbers. Block randomization was utilized, with random block sizes of four. To ensure allocation concealment, each child was given a serial number written on identical sheets of paper with the group to which they were assigned and placed inside opaque envelopes with their names. Trial independent personnel were assigned the task of preserving the envelopes and opening them only at the moment of intervention, so concealing the child’s group assignment from the outcome evaluator. Due to the study’s nature, the operator could not be blinded. However, the participants and statistician were blinded to the treatment groups, therefore, this clinical trial is double-blind.

### Calibration

The primary researcher underwent rigorous training and calibration for the application of ICDAS criteria [27] to diagnose dental caries in the study’s selected teeth and for the caries removal procedure. Initial training consisted of online resources detailing the ICDAS methodology [28], which was supplemented by practical clinical training involving the examination of 10 children. To evaluate intra-examiner reliability [22], 10% of the total sample size (10 children aged 8 to 10 years) were assessed, with a re-examination conducted after seven days. This process demonstrated excellent reliability, with Kappa values of 0.93 and 0.91. These children were not included in the study sample.

For the caries removal procedure, a single operator conducted the caries excavation on 10 children. The principal investigator, who was blinded to the specific technique used (Brix 3000, Papacárie Duo, or hand excavation), performed clinical evaluations to calibrate the operator. Evaluation involved the use of a blunt-tip probe (SS White Duflex, Rio de Janeiro, Brazil) to assess the texture of the remaining dentin and verify a vitreous appearance, indicative of complete caries removal [29]. A new excavator was used for each tooth [30]. Caries removal was considered complete when consensus was reached between the operator and the examiner. Intra-examiner reliability for this procedure was also assessed with the Kappa statistic, yielding an excellent value of 0.92.

For the radiographic evaluation of periapical lesions incidence and apical closure, the main examiner (PHH) underwent a calibration process with the study supervisor (DMT). A total of 30 randomly selected radiographs were independently assessed by both examiners. Discrepancies were discussed immediately to reach consensus and ensure consistency in interpretation. Inter-examiner reliability was calculated using the Kappa statistic, resulting in a value of 0.85, indicating strong agreement.

### Effect modification/cofounders

Children’s socioeconomic status, food, and oral health practices (As reported by their guardians) were assessed at the beginning using the Arabic version of the WHO questionnaire assessing the children’s oral hygiene habits [31].

### Baseline examination

At baseline, the researcher provided oral hygiene instructions to each study participant and informed them about the necessity of maintaining good oral hygiene and a proper diet guidance [22]. Additionally, pre-enrollment periapical X-ray radiographs were taken for each tooth to confirm that the carious lesion depth was not near the pulp.

### Intervention

Prior to and after each caries removal procedure, carious lesions in participants were sampled for microbiological examination. To prevent plaque bacteria contamination, participants rinsed with water, and the outer surface of the lesion was cleaned with a water spray [9].

#### Baseline sample

A microbiological sample was collected from the carious dentin at the floor of the cavity using a sharp, sterile spoon excavator before caries removal.

#### In the CMCR group

**Fig. 1:**
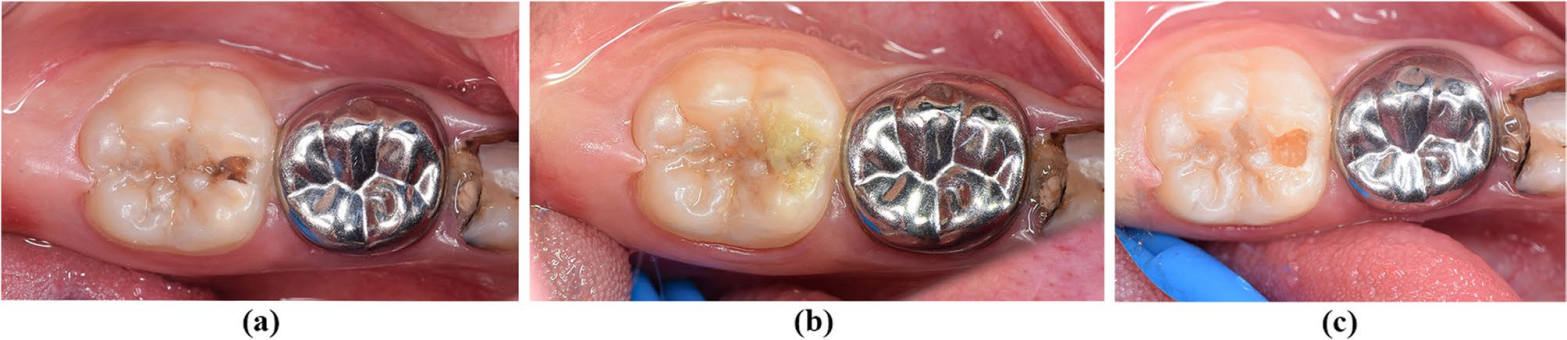
Clinical aspect of cavity before and after removal of carious tissue with Brix 3000. **a** Clinical view of the cavity prior to caries removal, (**b**) Application of Brix 3000, (**c**) 532 Cavity after the removal of carious tissue using Brix 3000

**Fig. 2:**
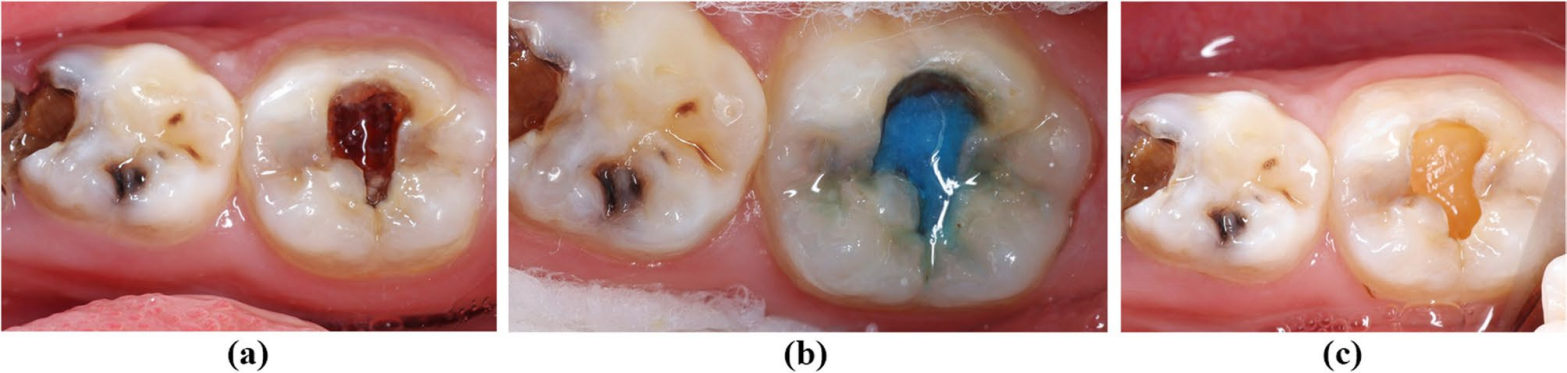
Clinical aspect of cavity before and after removal of carious tissue with Papacárie Duo. **a** Clinical view of the cavity prior to caries removal, (**b**) Application of Papacárie 529 Duo, (**c**) Cavity after the removal of carious tissue using Papacárie Duo

Cotton rolls and a saliva ejector were used to partially isolate the teeth then either Brix3000 gel (test group I) (Brix S.R.L, Carcarañá, Santa Fé, Argentina) (Fig. 1) or Papacárie Duo (test group II) (Fig. 2) (Formula and Acao, Sao Paulo, Brazil) was applied to the tooth’s carious lesion and allowed to sit for 1–2 min, as instructed by the manufacturer and described in the previous studies [20, 32]. This produced the carious dentin to be softened, which was scraped with a hand excavator (Nordent Manufacturing Inc. USA). This procedure was carried out two or three more times until the dentin exhibits a minor resistance and there is no tug-back sensation when an exploratory probe is pressed into the dentin [33]. At that point, the chemo-mechanical agent was no longer applied. The CMCR agent’s non-turbid appearance was the basis for the visual test used to evaluate full caries excavation.

#### In the control group

The study utilized ART with selective caries removal by hand instruments. No chemical agents were used, and the caries tissue was removed manually. ART was chosen as the comparison method due to its minimal discomfort and its ability to remove carious tissue without the need for anesthesia. Hand excavation was performed to remove the carious tissue from the cavity by using a sterile sharp hand excavator (Nordent Manufacturing Inc. USA). The cavity was determined to be caries-free according to visual and tactile clinical criteria [33], which is well-supported by the literature for its reliability and validity [34, 35].

Local anesthesia was administered to any participant who experienced pain, as deemed necessary, across all study groups.

#### Second sample

After complete caries removal in each group, a second microbial sample was collected as described previously.

Following the complete removal of dental decay, Dentin Conditioner (GC Corporation, Tokyo, Japan) was applied for 20 s using a micro-applicator. The cavity was then thoroughly rinsed with water. Excess water was carefully removed with an air syringe to achieve a dry surface without desiccation, maintaining a moist and glistening appearance.

The restoration was carried out using EQUIA Forte HT Fil (GC Corporation, Tokyo, Japan), a glass ionomer known for its enhanced mechanical properties, which renders it suitable for posterior restorations and preferred for minimally invasive dental procedures [36]. Pressure was applied to the glass ionomer with a gloved finger for one minute. After verifying the occlusion and removing any excess material, EQUIA Forte Coat (GC Corporation, Tokyo, Japan) was immediately applied with a disposable micro-tip applicator and light-cured using a light-emitting diode curing unit (1000 mW/cm²) for 20 s.

### Evaluations

#### Evaluation of time required to perform procedure

The duration required for complete caries removal was recorded for each method using a stopwatch. In the CMCR groups, timing began with the application of the gel, whereas in the hand excavation group, timing started at the initiation of excavation, as no gel was used. The stopwatch was stopped when all carious dentin had been removed from the cavity.

#### The participants’ pain assessment

Subjective pain levels were assessed after caries removal using the Wong-Baker FACES pain rating scale (PRS) [37]. This scale features a series of facial expressions depicting varying degrees of happiness and pain, with scores ranging from 0 to 5, where 0 represents no pain and 5 indicates severe pain. This scale was chosen due to its proven reliability, reproducibility, affordability, child-friendly design, and its effectiveness in capturing pain perception especially in children aged 8–10 years, who are at the concrete operational stage of cognitive development and are capable of accurately reporting their pain levels [38, 39].

To ensure accurate use of the scale, each child received training before the assessment. The training involved modeling the use of the scale and asking each participant to recall a recent painful experience, then select the facial expression that best represented their discomfort.

#### Microbiological assessment

Each dentin sample was placed in a sterile tube with 1 ml of saline and transported to the microbiology lab within 1–2 h. Sample weight was calculated by subtracting the weight of the transport medium and sterile container from the total weight, and bacterial counts were expressed as CFU/mg of dentin. Samples were collected before and after caries removal to assess reductions in *S. mutans* and *Lactobacilli*. After vortexing for 30 s, samples were serially diluted and plated on Mitis Salivarius agar for *S. mutans* and Rogosa agar for *Lactobacilli*. *S. mutans* plates were incubated anaerobically at 37 °C with 10% CO₂ for 72 h, while *Lactobacilli* were incubated aerobically at 37 °C for 48 h. Colonies were identified by morphology and counted, with CFU/ml calculated using the formula:

$$\mathrm{CFU}/\mathrm{ml}\;=\frac{\mathrm n^\circ\mathit\;\mathrm{of}\;\mathrm{colonies}\;\times\;\mathrm{dilution}\;\mathrm{factor}}{\mathrm{Volume}\;\mathrm{taken}\;\mathrm{in}\;\mathrm{ml}}$$

#### Clinical evaluation

Clinical assessments were conducted at 3-, 6-, and 12-month intervals to evaluate several parameters: (a) changes in tooth color and the presence of fistulas, (b) postoperative pain, and (c) restoration success. Restoration success was determined based on the criteria outlined by Phantumvanit et al. (1996) [40]. Restorations were classified as successful if they received scores of 0, 1, or 7, and as failures if they received scores of 2, 3, 4, 5, or 8. Scores of 6 were excluded from the analysis. (Table S1)

#### Radiographic evaluation

A periapical radiograph was taken immediately following the clinical procedure to serve as the baseline for comparison with follow-up radiographs. Additional radiographs were obtained at 6- and 12-months during follow-up visits to corroborate the clinical evaluations and assess the long-term outcomes [41]. These radiographs were acquired using the long cone paralleling technique with film holders to minimize distortion [42]. Cases that showed signs of pulp involvement, indicated by radiolucent images or periapical lesions, were deemed unsuccessful. Conversely, treatments were considered successful if apical closure and root growth continued without any pathological findings.

#### Oral health quality of life questionnaire

Oral health-related quality of life (OHRQoL) was evaluated using the Arabic validated version of the Child Perceptions Questionnaire (CPQ8–10) [43], at baseline, one-week post-treatment, and at 6- and 12-month intervals. The CPQ8–10 comprises 25 items across four domains: oral symptoms, functional limitations, emotional well-being, and social well-being. Children self-reported their responses on a 5-point Likert scale (0–4 per item), with total scores ranging from 0 to 100, where higher scores indicate poorer OHRQoL.

### Statistical analysis

Data analysis was performed using IBM SPSS version 23 (IBM Corp., Armonk, USA). The distribution normality was assessed with the Kolmogorov-Smirnov and Shapiro-Wilk tests. Differences in the duration of caries removal, pain reactions and bacterial counts and percent reduction in bacteria between the groups were analyzed using the Kruskal-Wallis test, and pairwise comparisons conducted using the Dunn-Bonferroni test. To evaluate differences in clinical and radiographic outcomes within and between groups over time, both the Chi-square test and Related Samples Cochran’s Q test were employed. Oral Health-Related Quality of Life (OHRQoL) was evaluated between and within the groups pre- and post-intervention and during follow-up using Kruskal-Wallis and Friedman tests. A significance level was set at (*P* < 0.05).

## Results

**Fig. 3:**
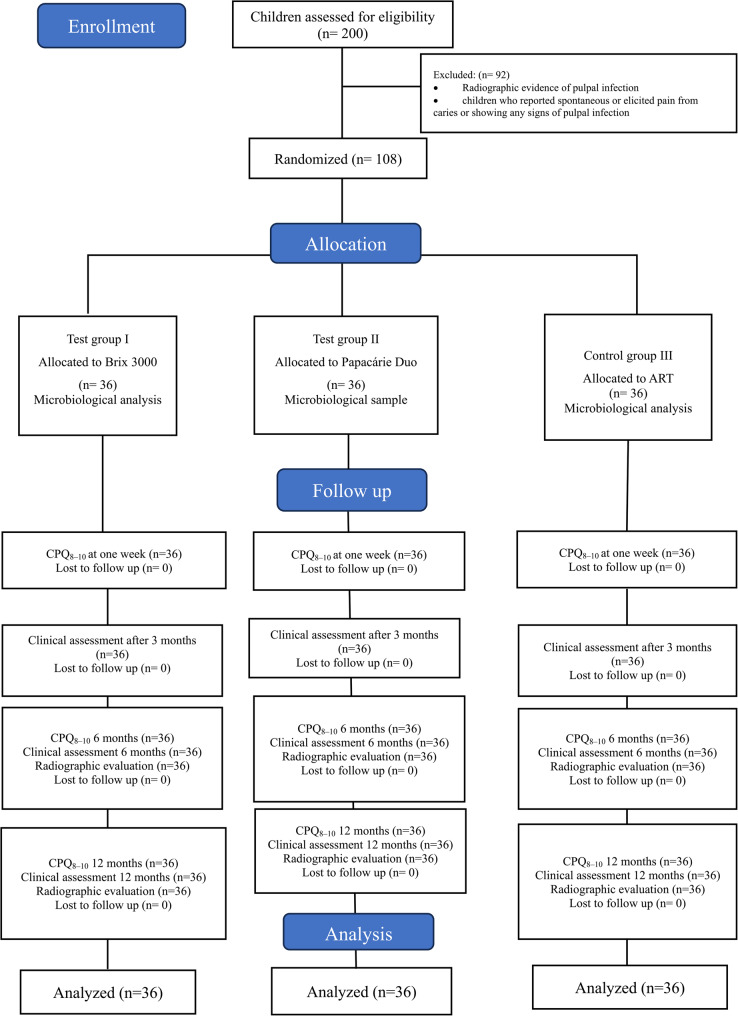
CONSORT flow diagram of the study design

A total of 200 healthy children with one occlusal carious lesion in their first permanent molar were initially examined. Ninety-two children were excluded from the study for reasons including spontaneous or elicited pain from caries, signs of pulpal infection, swelling, abscess, and failure to meet the inclusion criteria of cooperation (Frankl 3 and 4). A total of 108 children were enrolled in the study (Fig. 3).

The mean (SD) ages of the participants were 8.83 (0.78) years for those treated with Brix 3000, 8.75 (0.77) years for those treated with Papacárie Duo, and 8.97 (0.80) years for those treated with hand excavation. The majority of participants were female (*N* = 58, 54%). According to the WHO questionnaire results, there were no statistically significant differences between the groups at baseline in terms of age, sex, tooth location, sugar scores, or mother’s education level confirming sample homogeneity (*P* > 0.05) (Table S2).

### The required time of complete dental caries removing

Between-group comparisons revealed that the 8.75 (0.77) years for those treated with Papacárie Duo, and 8.97 (0.80) years for those treated with hand excavation required significantly less time for caries removal compared to both Brix 3000 (*P* < 0.001) and Papacárie Duo (*P* < 0.001), with no significant difference observed between Brix 3000 and Papacárie Duo (*P* = 1). (Table 1) (Table S3).

**Table 1:** Time of caries removal and pain reaction among the study groups

Groups	Brix 3000 *n* = 36	Papacárie Duo *n* = 36	ART *n* = 36	*P*^±^ value
Time (minutes) Median (IQR)	11.3(3.01) ^a^	12.1(2.03) ^a^	5.3(2.1) ^b^	< 0.001*
Wong-Baker Scale Mean Rank	50.35 ^a^	42.15 ^a^	71.00 ^b^	< 0.001*

± Kruskal-Wallis test

* Statistically significant at P < 0.05, a and b: different letters denote statistically significant differences between the groups

### Pain sensation assessment

Between-group comparisons revealed a statistically significantly higher pain score in the control group compared to both the Brix 3000 (*P* = 0.003) and Papacárie Duo (*P* < 0.001) groups. However, there was no significant difference in pain scores observed between the Brix 3000 and Papacárie Duo groups (*P* = 0.56). (Table 1) (Table S4).

### Microbiological assessment

Within-group comparisons showed a statistically significant reduction in the median log counts (CFU/mL) of *S. mutans* and *Lactobacilli* between baseline and post-intervention in all groups (*P* < 0.001). Brix 3000 and Papacárie Duo demonstrated a more pronounced antimicrobial effect than control group.

Between-group comparisons revealed that both CMCR agents achieved significantly greater percent reductions in *S. mutans* compared to hand excavation (*P* < 0.001). Although no statistically significant difference was observed between the two CMCR agents (*P* > 0.05), Papacárie Duo showed slightly greater reduction than Brix 3000 (Table 2).

**Table 2:** Comparison of *S. mutans* counts in lesion samples among study groups

		Brix 3000 *n* = 36	Papacarie Duo *n* = 36	ART *n* = 36	*P* value^±^
		Median (IQR)			
Pre-intervention	Count	7.85 (33.02) x10^4^	3.45 (21.46) x10^4^	3.50 (12.69) x10^4^	0.834
	Log	4.857 (2.16)	4.54 (1.25)	4.54 (1.23)	
Post-intervention	Count	0.0027 (0.009) x 10^3 a^	0.0018 (0.009) x10^3 a^	3.00 (9.79) 10^3 b^	< 0.001*
	Log	0.57 (1.00)	0.45 (1.00)	3.48 (1.99)	
P^¶^		< 0.001*	< 0.001*	< 0.001*	
% Reduction		83.64 (33.84) ^a^	90.06 (24.34) ^a^	27.31 (31.73) ^b^	< 0.001*

± Kruskal-Wallis test, ¶ Wilcoxon Sign Rank

* Statistically significant at P < 0.05, a and b: different letters denote statistically significant differences

Similarly, shows a significantly greater percent reduction in *Lactobacilli* for Brix 3000 and Papacárie Duo compared to hand excavation (*P* < 0.001). While the difference between the two CMCR agents was not statistically significant (*P* > 0.05), Papacárie Duo demonstrated higher bacterial reduction than Brix 3000 (Table 3).

**Table 3:** Comparison of *Lactobacilli* counts in lesion samples among study groups

		Brix 3000 *N* = 36	Papacarie Duo *N* = 36	ART *N* = 36	*P*^±^
		Median (IQR)			
Pre-intervention	Count	3.40 (9.89) x 10^5^	2.95 (2.79) x 10^5^	2.1 (3.19) x10^5^	0.078
	Log	5.53 (1.85)	5.47 (0.95)	5.32 (1.20)	
Post-intervention	Count	0.0054 (0.062) x10^3 a^	0.0054 (0.072) x10^3 a^	2.40 (22.82) x10^3 b^	< 0.001*
	Log	0.80 (1.78)	0.72 (1.86)	3.38 (2.49)	
P^¶^		< 0.001*	< 0.001*	< 0.001*	
% Reduction		81.94 (38.51) ^a^	86.91 (34.34) ^a^	33.50 (22.30) ^b^	< 0.001*

± Kruskal-Wallis test, ¶ Wilcoxon Sign Rank

* Statistically significant at P < 0.05, a and b: different letters denote statistically significant differences

### Clinical follow up

#### Tooth color alternation and fistulas

Regarding tooth color alteration and fistula presence, after 3 months, only one child in the control group showed a fistula. After 12 months, another participant in the same group developed a fistula. In contrast, no participants in the Brix 3000 or Papacárie Duo groups showed any tooth color alteration or fistula throughout the study period. However, there was no significant difference between the groups (*P* > 0.5). (Table 4)

**Table 4:** Tooth color alternation, fistula presence and post operative pain evaluation at the 3, 6 and 12 months follow up between and within the study groups

			Brix 3000 (*n* = 36)	Papacarie Duo (*n* = 36)	ART (*n* = 36)	*p*^†^ value^1^
			n (%)			
Tooth color alternation and fistula	3 months	Yes	0 (0%)	0 (0%)	1 (2.8%)	0.40
		No	36 (100%)	36 (100%)	35 (97.2%)	
	6 Months	Yes	0 (0%)	0 (0%)	1 (2.85%)	0.40
		No	36 (100%)	36 (100%)	35 (97.2%)	
	12 Months	Yes	0 (0%)	0 (0%)	2 (5.6%)	0.13
		No	36 (100%)	36 (100%)	34 (94.4%)	
	***p***^**‡**^ value^2^		1.00	1.00	0.37	
Post operative Pain	3 months	Yes	35 (97.2%)	36 (100%)	33 (91.7%)	0.009*
		No	1 (2.8%)	0 (0%)	3 (8.3%)	
	6 Months	Yes	34 (94.4%)	36 (100%)	30 (83.3%)	0.009*
		No	2 (5.6%)	0 (0%)	3 (8.3%)	
	12 Months	Yes	34 (94.4%)	36 (100%)	30 (83.3%)	0.009*
		No	2 (5.6%)	0 (0%)	6 (16.7%)	
	***p***^**‡**^ value^2^		1.00	1.00	1.00	

* Statistically significant at P < 0.05, † Chi-square test, ‡ Related Samples Cochrane’s Q test

P^1^ value for tooth color alternation and fistula, and pain between the study groups at each follow up point

P^2^ value for tooth color alternation and fistula, and pain within each group across time

#### Post operative pain

Throughout one year of clinical follow-up, the Papacárie Duo group showed that all treated molars had no pain. Meanwhile, in the Brix 3000 group, only one child complained of pulp sensitivity after 3 months. In the control group, 6 participants reported pain after 3 months, with significant differences between the groups (*P* = 0.009). (Table 4)

#### Restoration success

At the three-month evaluation, the restoration success rates were 97.2%, 100.0%, and 91.7% for the Brix 3000, Papacárie Duo, and control groups, respectively, with no significant difference between the three groups (*P* = 0.16). At the six-month and twelve-month evaluations, the restoration success rates were 94.4%, 100%, and 83.3% for Brix 3000, Papacárie Duo, and hand excavation, respectively, with statistically significant differences (*P* = 0.02). (Table 5)

**Table 5:** Restoration success at the 3, 6 and 12 months follow up between and within the study groups

			Brix 3000 (*n* = 36)	Papacarie Duo (*n* = 36)	ART (*n* = 36)	*p*^†^ value^1^
			*n* (%)			
Restoration success	3 months	Yes	35 (97.2%)	36 (100%)	33 (91.7%)	0.16
		No	1 (2.8%)	0 (0%)	3 (8.3%)	
	6 Months	Yes	34 (94.4%)	36 (100%)	30 (83.3%)	0.02*
		No	2 (5.6%)	0 (0%)	3 (8.3%)	
	12 Months	Yes	34 (94.4%)	36 (100%)	30 (83.3%)	0.02*
		No	2 (5.6%)	0 (0%)	6 (16.7%)	
***p***^**‡**^ value^2^			0.05	1.00	0.05	

* Statistically significant at P < 0.05, † Chi-square test, ‡ Related Samples Cochrane’s Q test

P^1^ value for restoration success between the study groups at each follow up point

P^2^ value for restoration success within each group across time

### Radiographic follow up

#### Apical closure

The apical closure rates at the 6-month evaluation were 97.2%, 100.0%, and 88.9% for the Brix 3000, Papacárie Duo, and hand excavation groups, respectively, with no significant differences between the groups (*P* = 0.06). At the 12-month evaluation, the apical closure rates were 97.2%, 100%, and 83.3% for the Brix 3000, Papacárie Duo, and hand excavation groups, respectively, with statistically significant differences (*P* = 0.009). (Table 6)

**Table 6:** Apical closure and periapical lesion evaluation at the 6 and 12 months follow up between and within the study groups

			Brix 3000 (*n* = 36)	Papacarie Duo (*n* = 36)	ART (*n* = 36)	*p*^†^ value^1^
			n (%)			
Apical closure	6 Months	Yes	35 (97.2%)	36 (100%)	32 (88.9%)	0.06
		No	1 (2.8%)	0 (0%)	4 (11.1%)	
	12 Months	Yes	35 (97.2%)	36 (100%)	30 (83.3%)	0.009*
		No	1 (2.8%)	0 (0%)	6 (16.7%)	
	***p***^**‡**^ value^2^		1.00	1.00	0.15	
Periapical lesion	6 Months	Yes	0 (0%)	0 (0%)	2 (5.6%)	0.13
		No	36 (100%)	36 (100%)	34 (94.4%)	
	12 Months	Yes	0 (0%)	0 (0%)	5 (13.9%)	0.005*
		No	36 (100%)	36 (100%)	31 (86.1%)	
	***p***^**‡**^ value^2^		1.00	1.00	0.08	

* Statistically significant at P < 0.05, † Chi-square test, ‡ Related Samples Cochrane’s Q test

P^1^ value for apical closure and periapical lesion between the study groups at each follow up point

P^2^ value for apical closure and periapical lesion within each group across time

#### Absence of periapical lesion

Regarding the incidence of periapical lesions, it was found that only two participants in the control group complained of periapical lesions at six months, with reported pain, which was recorded as part of the post-operative clinical evaluations. There was no significant difference between the three groups (*P* = 0.13). In contrast, none of the treated participants in the Brix 3000 or Papacárie Duo groups showed periapical lesions during the 12 months of follow-up, with significant differences between the groups (*P* = 0.005). (Table 6)

### Oral Health-Related quality of life

Within-group comparison, CPQ8–10 scores showed significant improvement throughout follow up period (*P* < 0.0001). Between group comparison there were no significant differences at baseline, after one week or 12 months post-treatment (*P* = 0.103, 0.234, 0.066, respectively). Only scores at 6 months demonstrated a significant difference between the groups (*P* = 0.008) (Table 7). Results showed statistically differences between ART and Papacárie Duo (*P* = 0.012), and between ART and Brix 3000 (*P* = 0.046). while there was no significant difference between Papacárie Duo and Brix 3000 (*P* = 1) (Table S5).

**Table 7:** Comparison of total OHQoL scores among the study groups at different follow up intervals

		Brix 3000	Papacarie duo	ART	*p*^¥^ value
Baseline	Mean ± SD	10.33 ± 3.66	9.39 ± 3.92	11.92 ± 4.94	0.103
	Median (IQR)	10.00 (1.80)	10.00 (6.00)	10.00 (4.50)	
1 st week	Mean ± SD	0.33 ± 1.69	0.00 ± 0.00	0.42 ± 1.40	0.234
	Median (IQR)	0.00 (0.00)	0.00 (0.00)	0.00 (0.00)	
6 months	Mean ± SD	0.06 ± 0.33	0.00 ± 0.00	1.17 ± 3.08	0.008*
	Median (IQR)	0.00 (0.00) ^a^	0.00 (0.00) ^a^	0.00 (0.00) ^b^	
12 months	Mean ± SD	0.06 ± 0.33	0.00 ± 0.00	0.25 ± 0.73	0.066
	Median (IQR)	0.00 (0.00)	0.00 (0.00)	0.00 (0.00)	
*p*^§^ value		**< **0.001*	**< **0.001*	**< **0.001*	
*Pairwise comparisons*		***p***_1_ < 0.001* ***P***_2_ < 0.001* ***P***_3_ < 0.001*	***p***_1_ < 0.001* ***P***_2_ < 0.001* ***P***_3_ < 0.001*	***p***_1_ < 0.001* ***P***_2_ < 0.001* ***P***_3_ < 0.001*	

* Statistically significant at p < 0.05, ¥ Kruskal-Wallis test, § Friedman test. different lowercase superscript letters denote statistically significant difference between groups

p1 comparison between baseline and 1 st week

p2 comparison between baseline and 6 months

p3 comparison between baseline and 12 months

## Discussion

Based on the results of the present study, the null hypothesis was rejected. Using CMCR agents for caries removal resulted in a significantly more operating time, less pain score, enhanced antimicrobial activity, higher clinical and radiographic success with improved OHRQoL than ART alone.

By comparing the three groups, the results revealed the longer time was required for complete caries removal with CMCR compared to hand excavation. This could be explained by the CMCR agents’ method of action which require to be left undisturbed for 2 min in the one time of application. Moreover, according to the lesion size and consistency, whether it is soft or medium or hard, reapplication of these agents usually was required to ensure complete caries removal. These findings are in agreement with other studies [3, 21, 44, 45].

While hand excavation was the faster method of caries removal, it was associated with greater pain and discomfort compared to CMCR. It is noted that removing infected dentin usually does not cause pain; however, discomfort can arise if the removal process extends toward sound, intact dentin [21, 44]. Therefore, ART approach alone can cause discomfort as it involved removing both both affected and healthy dentin. In contrast, papain-based agents specifically target and remove only the soft, infected dentin, thereby preserving the affected dentin. Papain achieves this by breaking down collagen without affecting the underlying dentin. On the other hand, ART with hand excavators may penetrate hard dentin, potentially expose more dentinal tubules and cause more discomfort [7, 19]. Thus, despite the longer operative time with CMCR agents, this did not negatively impact the participants’ overall experience, aligning with the findings of Gupta et al. [46].

Treatment with Brix 3000 resulted in significant reductions in both *S. mutans* and *Lactobacilli*, consistent with the findings of Inamdar et al. and Kandil et al. [47, 48]. which can be attributed to its high concentration of papain (30,000 U/mg) and the antimicrobial effect of toluidine blue. Similarly, Papacárie Duo demonstrated a significant reduction in both bacterial species, in line with results reported by Reddy et al. and Balachandran et al. [9, 49]. Its effectiveness is associated with a substantial papain concentration (6,000 U/mg) and the antibacterial action of chloramine. Although no statistically significant difference was observed between Brix 3000 and Papacárie Duo, the latter showed slightly greater efficiency. Furthermore, between-group comparisons revealed that both CMCR agents significantly outperformed hand excavation alone in reducing bacterial counts, supporting Cardoso et al.‘s findings on the superior antimicrobial efficacy of papain-based methods compared to conventional techniques [50].

Throughout the clinical follow-up, within-group analyses revealed no significant changes across the various time intervals. The control group experienced occasional instances of fistula development at both 3 and 12 months, whereas neither the Brix 3000 nor the Papacárie Duo groups reported such occurrences. Postoperative pain assessments indicated that the Papacárie Duo group consistently demonstrated an absence of pulp sensitivity throughout the follow-up period. In contrast, isolated instances of pain sensation were observed in both the Brix 3000 and control groups. Restoration success rates were consistently high and stable across all groups throughout the follow-up period. There were no significant variations in apical closure rates or periapical lesions incidence over time within each group. These results align with the findings of de Amoriom et al. [51], who reported similar survival rates for hand excavation. Similarly, El Wazeer et al. [52] and Duman et al. [16], observed comparable clinical success with Brix 3000, while Motta et al. [24] reported on the efficacy of Papacárie Duo.

Comparison between groups revealed significant differences in the clinical and radiographic outcomes. The control group exhibited a higher incidence of post-operative pulp pain. Although instances of tooth color alteration and fistulas were observed in the control group, these did not differ significantly from the CMCR groups. At six and twelve months, Papacárie Duo group demonstrated the highest success rates, followed by the Brix 3000 group, while the control group showed the lowest success rates. Radiographically, the Papacárie Duo group demonstrated superior apical closure rates at the 12-month evaluation compared to both the Brix 3000 and control groups. The hand excavation group had the lowest apical closure rates and a higher incidence of periapical lesions compared to the other two groups. Both Brix 3000 and Papacárie Duo have been shown to effectively eliminate the smear layer and promote mineral deposition around dentinal tubules [53, 54]. These factors likely contributed to the superior clinical success observed. In contrast, hand excavation without the use of chemical agents may reduce its precision and effectiveness, as it often leaves a smear layer and removes more enamel, potentially compromising restoration adhesion and contributing to its lower success rate [55].

Notably, there are no published studies directly comparing the clinical effects of Brix 3000 and Papacárie Duo with hand excavation. The high success rates of Papacárie Duo are consistent with findings by Cardoso et al. [50], who attributed its superior performance to its effective antibacterial properties. These properties are likely due to its chloramine content, which is known for its strong antimicrobial action, as well as its lower viscosity, which allows the agent to penetrate dentinal tubules more effectively. The combination of these factors enables Papacárie Duo to achieve a deeper and more uniform bacterial elimination compared to other CMCR agents as Brix 3000. By eliminating bacteria within the dentin, Papacárie Duo not only aids in caries removal but also reduces the bacterial load within the cavity, contributing to a cleaner and safer environment for restoration. This enhanced antibacterial effect could reduce the risk of postoperative infection, improving the long-term success of the restorations.

In the current study, there was a significant reduction in CPQ8–10 scores across all three study groups following treatment. This finding aligns with Aimée et al. [56], which indicates that untreated dental caries has a greater impact on quality of life compared to post-treatment conditions. Improvements in CPQ8–10 scores were closely linked to clinical success, suggesting that enhancements in OHRQoL are more related to the effectiveness of the restoration rather than receiving the treatment. Although CMCR was significantly more effective than ART in the short term, the lack of significant differences at 12 months suggests that both treatments may offer comparable long-term effect on OHRQoL.

In conclusion, the results of this study highlight the superior performance of CMCR agents compared to hand excavation in terms of restoration longevity, pain reduction, and treatment comfort. However, several limitations should be acknowledged. These include the lack of standardization in the application of CMCR agents, the high cost and short shelf life of Papacárie Duo, and the inclusion of only cooperative children (Frankl 3 and 4), which may limit the generalizability of the findings. Additionally, the operator was not blinded to treatment allocation due to the distinct visual and physical characteristics of the caries removal agents, potentially influencing the assessment of the intervention.

Future research should address these limitations by including a more diverse range of pediatric patients, with varying levels of cooperation and across different age groups, to improve the applicability of the findings.

## Conclusions

Based on the current study’s findings, these conclusions were made.

1. Papacárie Duo achieved superior clinical success rate than Brix 3000 and selective caries removal by hand excavation alone.
2. ART enhanced by CMCR methods such as Brix 3000 and Papacárie Duo provides an effective alternative to conventional caries removal methods, particularly for children who are afraid of drills and burs.
3. Brix 3000 and Papacárie Duo demonstrated superior antimicrobial efficacy against *S. mutans*, and *Lactobacilli* compared to ART alone, with both agents showing comparable performance.
4. Chemo-mechanical caries removal agents can improve oral health-related quality of life in children with caries.

## Supplementary Information

Supplementary Material 1.

## Data Availability

Patients’ records at the Pediatric Dentistry Department, Faculty of Dentistry, Alexandria University, contain the datasets created and analyzed for the current study and were used under license for this study. The public accessibility of such data is subject to restrictions. However, data from the corresponding author are available upon reasonable request and with permission from the Pediatric Dentistry Department, Faculty of Dentistry, University of Alexandria.

## Abbreviations

ART: Atraumatic restorative treatment
CMCR: Chemo-mechanical caries removal
CFU: colony-forming units
CPQ8–10: Child perceptions questionnaire
IRB: Institutional review boards
ICDAS: International detection and assessment system
OHRQoL: Oral health-related quality of life
PRS: Wong-Baker FACES pain rating scale
S. mutans: Streptococcus mutans
