# Supplementary material for: One-year clinical and radiographic evaluation of young permanent molars treated with brix 3000 vs. papacárie duo: a randomized controlled clinical trial
Source: BMC Oral Health. 2025 Sep 23;25:1421. doi: 10.1186/s12903-025-06715-7 (PMC12459045; doi:10.1186/s12903-025-06715-7)
Supplement: Supplementary file 1 — Supplementary Material 1. [file 12903_2025_6715_MOESM1_ESM.docx]

| **Codes** | **Description** |
| --- | --- |
| **0** | Present, in a good condition |
| **1** | Present, slight marginal defect, no repair needed |
| **2** | Present, marginal defects 0.5-1.0 mm, repair needed |
| **3** | Present, marginal defects < 1.0 mm, repair needed |
| **4** | Not present, restoration partially or completely missing |
| **5** | Not present, restoration replaced by other restoration |
| **6** | Tooth missing, exfoliated or extracted |
| **7** | Present, slight wear, no repair needed |
| **8** | Present, wear> 0.5mm, repair needed |

**Table S1: Evaluation scores criteria of atraumatic restorative treatment proposed by Phantumvanit et al**.

**Table S2: Socio-demographic characteristics, oral health behaviors and clinical features in children included in the study at baseline (n =108)**

| **Variables** | | **Groups** | | | |
| --- | --- | --- | --- | --- | --- |
|  |  | **Brix 3000**  n=36 | **Papacarie Duo**  n=36 | **ART**  n=36 | **P value** |
| **Child age** | Median (SD)  Min- Max | 8.83) 0.78)  8-10 | 8.75) 0.77(  8-10 | 8.97(0.8(  8-10 | 0.5 |
| **Child sex** | Male: n (%) | 17(47.2%) | 13(36.1%) | 20 (55.6%) | 0.25 |
|  | Female: n (%) | 19(52.8%) | 23(63.9%) | 16(44.4%) |  |
| **Mother’s education** | Primary school or less: n (%) | 13 (36.1%) | 11 (30.6%) | 12 (33.3%) | 0.09 |
|  | Middle school: n (%) | 13 (36.1%) | 9 (25%) | 18 (50%) |  |
|  | High school and higher: n (%) | 10 (27.8%) | 16 (44.4%) | 6 (17.7%) |  |
| **Father’s education** | Primary school or less: n (%) | 8 (22.2%) | 7 (19.4%) | 6 (16.7%) | 0.29 |
|  | Middle school: n (%) | 10 (27.8%) | 9 (25%) | 17 (47.2%) |  |
|  | High school and higher: n (%) | 18 (50%) | 20 (55.6%) | 13 (36.1%) |  |
| **Sugar score** | Median (IQR) | 18(2) | 18(3.5) | 19(3) | 0.05 |
| **Dental visits in the last year** | Yes: n (%) | 21(58.3%) | 17(47.2%) | 24(66.70%) | 0.53 |
|  | No: n (%) | 12(33.3%) | 16(44.4%) | 9(25.0%) |  |
|  | Don’t Know: n (%) | 3(8.3%) | 3(8.3%) | 3(8.3%) |  |
| **Toothbrushing once or more daily** | Yes: n (%) | 10(27.8%) | 7(19.4%) | 8(22.2%) | 0.69 |
|  | No: n (%) | 26(72.2%) | 29(80.6%) | 28(77.8%) |  |
| **Tooth Location** | Upper: n (%) | 11(30.6%) | 12(33.3%) | 10(27.8%) | 0.88 |
|  | Lower: n (%) | 25(69.4%) | 24(66.7%) | 26(72.2%) |  |

**Table S3: Pairwise comparisons among the study groups for the time of caries removal**

|  | **Compared to** | **P** ^¥^ **value** |
| --- | --- | --- |
| **ART** | Papacarie Duo | <0.001* |
|  | Brix 3000 | <0.001* |
| **Papacarie Duo** | Brix 3000 | 1 |

¥ DUN-bonferroni, *****Statistically significant at ***p <0.05***

**Table S4: Pairwise comparisons among the study groups for the Pain reaction**

|  | **Compared to** | **P** ^¥^ **value** |
| --- | --- | --- |
| **ART** | Papacarie Duo | <0.001* |
|  | Brix 3000 | 0.003* |
| **Papacarie Duo** | Brix 3000 | 0.56 |

¥ DUN-bonferroni, *****Statistically significant at ***p <0.05***

**Table S5: Pairwise comparisons among the study groups for the OHQOL at 6 months**

| **Groups** | **Compared to** | **P ^¥^ value** |
| --- | --- | --- |
| ART | Papacarie duo | 0.012* |
|  | **Brix 3000** | 0.046* |
| Papacarie duo | **Brix 3000** | 1.00 |

¥ DUN-bonferroni, * Statistically significant at *p* <0.05
